# Sequential Open-Label Study of the Safety, Tolerability, and Pharmacokinetic Interactions between Dihydroartemisinin-Piperaquine and Mefloquine in Healthy Thai Adults

**DOI:** 10.1128/AAC.00060-19

**Published:** 2019-07-25

**Authors:** Borimas Hanboonkunupakarn, Rob W. van der Pluijm, Richard Hoglund, Sasithon Pukrittayakamee, Markus Winterberg, Mavuto Mukaka, Naomi Waithira, Kesinee Chotivanich, Pratap Singhasivanon, Nicholas J. White, Arjen M. Dondorp, Joel Tarning, Podjanee Jittamala

**Affiliations:** a Mahidol-Oxford Tropical Medicine Research Unit, Faculty of Tropical Medicine, Mahidol University, Bangkok, Thailand; b Department of Clinical Tropical Medicine, Faculty of Tropical Medicine, Mahidol University, Bangkok, Thailand; c Centre for Tropical Medicine and Global Health, Nuffield Department of Medicine, University of Oxford, Oxford, United Kingdom; d The Royal Society of Thailand, Bangkok, Thailand; e Department of Tropical Hygiene, Faculty of Tropical Medicine, Mahidol University, Bangkok, Thailand

**Keywords:** antimalarial agents, healthy subject, pharmacokinetics

## Abstract

Artemisinin-based combination therapies (ACTs) have contributed substantially to the global decline in Plasmodium falciparum morbidity and mortality, but resistance to artemisinins and their partner drugs is increasing in Southeast Asia, threatening malaria control. New antimalarial compounds will not be generally available soon. Combining three existing antimalarials in the form of triple ACTs, including dihydroartemisinin (DHA)-piperaquine + mefloquine, is a potential treatment option for multidrug-resistant Plasmodium falciparum malaria. In a sequential open-label study, healthy Thai volunteers were treated with DHA-piperaquine (120 to 960 mg), mefloquine (500 mg), and DHA-piperaquine + mefloquine (120 to 960 mg + 500 mg), and serial symptom questionnaires, biochemistry, full blood counts, pharmacokinetic profiles, and electrocardiographic measurements were performed. Fifteen healthy subjects were enrolled. There was no difference in the incidence or severity of adverse events between the three treatment arms. The slight prolongation in QTc (QT interval corrected for heart rate) associated with DHA-piperaquine administration did not increase after administration of DHA-piperaquine + mefloquine. The addition of mefloquine had no significant effect on the pharmacokinetic properties of piperaquine. However, coadministration of mefloquine significantly reduced the exposures to dihydroartemisinin for area under the concentration-time curve (−22.6%; 90% confidence interval [CI], −33.1, −10.4; *P* = 0.0039) and maximum concentration of drug in serum (−29.0%; 90% CI, −40.6, −15.1; *P* = 0.0079). Mefloquine can be added safely to dihydroartemisinin-piperaquine in malaria treatment. (This study has been registered at ClinicalTrials.gov under identifier NCT02324738.)

## Abstract (precis)

Artemisinin-based combination therapies (ACTs) have contributed substantially to the global decline in Plasmodium falciparum morbidity and mortality, but resistance to artemisinins and their partner drugs is increasing in Southeast Asia, threatening malaria control. New antimalarial compounds will not be generally available soon.

## INTRODUCTION

Artemisinin-based combination therapies (ACTs), which combine a potent and rapidly eliminated artemisinin derivative and a more slowly eliminated partner drug, have contributed significantly to the large global decline in Plasmodium falciparum morbidity and mortality (1–3). However, artemisinin resistance, which manifests as slow parasite clearance resulting from reduced susceptibility of ring-stage parasites to artemisinins, has emerged and spread in Southeast Asia (4–8). Artemisinin resistance is associated with mutations in the P. falciparum Kelch gene on chromosome 13 (*Kelch13*). The reduction in artemisinin sensitivity has left partner drugs within the ACTs exposed to larger numbers of parasites after the initial 3 days of treatment, and this has facilitated the selection and spread of artemisinin and partner drug resistance (9).

On the Myanmar-Thailand border the combination of artemisinin resistance and the reemergence of mefloquine resistance led to high failure rates following treatment of Plasmodium falciparum malaria with artesunate-mefloquine, forcing a change in policy (10). In Cambodia and southern Vietnam, artemisinin and piperaquine resistance have led to high rates of treatment failures after dihydroartemisinin (DHA)-piperaquine (11–15). The incidence of malaria has risen subsequently. New potent antimalarials, such as spiroindolones, imidazolopiperazines, and synthetic endoperoxides, are currently being tested in clinical trials, but their large-scale deployment is not expected to occur within the next 5 years (16, 17). There is therefore an urgent need to use existing antimalarials in novel ways to counter the threat of multidrug resistance in P. falciparum in the Greater Mekong Subregion (GMS). Recombining three existing antimalarials in the form of triple artemisinin-based combination therapies (TACTs) could be an important option for the treatment of multidrug-resistant Plasmodium falciparum malaria.

The matching pharmacokinetic profiles of piperaquine and mefloquine should ensure antimalarial activity and mutual protection from both partner drugs throughout a large part of the elimination phase of the partner drugs. In addition, the TACT DHA-piperaquine + mefloquine takes advantage of an inverse relationship of piperaquine and mefloquine resistance observed in field and laboratory studies (11–14, 18–21).

DHA-piperaquine is generally well tolerated, with most reported side effects being similar to and/or indistinguishable from malaria symptoms such as headache, gastrointestinal symptoms, and fatigue (22). Mefloquine has neuropsychiatric side effects, including headache, dizziness, and sleeping disturbances, and gastrointestinal side effects, including nausea, vomiting, abdominal pain, and diarrhea (23); nevertheless, it is generally well tolerated in the treatment of malaria. Piperaquine is known to cause dose-dependent electrocardiographic QT interval prolongation (24). This has led to concerns about its proarrhythmic potential, but a recent large systematic review indicated that the use of DHA-piperaquine does not increase the risk of sudden unexplained death (25). QT prolongation has been reported after the treatment of P. falciparum with artesunate-mefloquine, but this does not correlate with mefloquine drug levels; thus, it may be attributable to a malarial rather than a drug effect (26, 27).

In preparation for a multinational field trial on the safety, tolerability, and efficacy of the TACT DHA-piperaquine + mefloquine, we conducted a single-dose, sequential, open-label study in healthy volunteers to characterize the tolerability and the potential pharmacokinetic and pharmacodynamic interactions between dihydroartemisinin, piperaquine, and mefloquine in healthy Thai subjects.

## RESULTS

### Study participants.

Fifteen subjects (six males) was screened and subsequently enrolled in this study. The weight and body mass index (BMI) before the first dose of DHA-piperaquine ranged from 49.8 to 73.0 kg and 20.0 to 27.9 kg/m^2^, respectively (Table 1).

**TABLE 1:** Subject baseline demographics before drug administration

Variable^*a*^	Result^*b*^
Age (yr)	41.6 (24.3–51.3)
Male [*n*/*N* (%)]	6/15 (40)
Height (cm)	165 (144–178)
Body weight (kg)	61.6 (49.8–73.0)
Body mass index (kg/m^2^)	23.5 (2.6)
QTcF interval (ms)	423 (407–439)
QTcB interval (ms)	429 (407–458)
Plasma aspartate aminotransferase (U/liter)	17 (13–24)
Plasma alanine aminotransferase (U/liter)	13 (9–32)
Plasma alkaline phosphatase (U/liter)	51 (35–70)
Plasma total bilirubin (μmol/liter)	6.8 (1.7–11.5)
Plasma creatinine (μmol/liter)	70.7 (44.2–88.4)

a QTcF is Fridericia-corrected QT intervals and QTcB is Bazett-corrected QT intervals.

b Data are presented as medians (ranges) unless otherwise specified.

### Safety.

All subjects were included in the safety analyses. A total of 97 adverse events related to clinical symptoms were reported (Table 2). Nearly all were graded as mild (95/97, 97.9%), and two were moderate in severity. A mild rise in aspartate aminotransferase (AST) and alanine aminotransferase (ALT) was documented in one subject after DHA-piperaquine + mefloquine. Adverse events were attributed as possibly related to the study drugs in 72 out of 97 cases (74.2%), whereas the other events were judged as not related to the study drugs.

**TABLE 2:** Clinical adverse events and serious adverse events

Category	DHA-piperaquine^*a*^		DHA-piperaquine + mefloquine		Mefloquine	
	Possibly related to study drug	Not related to study drug	Possibly related to study drug	Not related to study drug	Possibly related to study drug	Not related to study drug
Central nervous system			9^*d*^	3	9	2
Cardiovascular		1^*b*^ (1 SAE)	3		1	
Gastrointestinal			20		14	2
Musculoskeletal			1	4	3	1
Dermatology			0	2^*e*^		
Infections		5^*c*^ (1 SAE)				
Sleeping disturbance			4	4	8	1
Total	0	6	37	13	35	6

a Subjects treated with DHA-piperaquine alone did not undergo a systematic symptom questionnaire.

b Unstable angina pectoris at day 12.

c One case of acute bronchitis, 2 of acute pharyngitis, 1 rickettsial infection, 1 viral infection of unknown origin.

d One neuropsychiatric reaction (anxiety, nausea, dizziness, and palpitations).

e One case of urticaria in subject with preexisting allergy to an unknown allergen.

In general, there was no difference in the incidence or severity of the study drug-related AEs when subjects were treated with DHA-piperaquine + mefloquine or mefloquine. The most common adverse events after treatment with DHA-piperaquine + mefloquine and mefloquine were mild to moderate dizziness, nausea, abdominal pain/discomfort, and disturbance of sleeping, which are all known side effects of mefloquine. One volunteer suffered from a moderate-severity neuropsychiatric reaction, as she reported anxiety, nausea, dizziness, and palpitations within 24 h after the administration of DHA-piperaquine and mefloquine. These complaints resolved completely by day 3. There were 2 serious adverse events (SAEs) during the previous studies in which DHA-piperaquine was administered. One subject had a rickettsial infection at day 24 after drug administration causing hospitalization. Another subject was hospitalized at day 12 after drug administration due to unstable angina pectoris. Both SAEs were assessed as not related to the study interventions.

### Cardiac effects.

**FIG 1:**
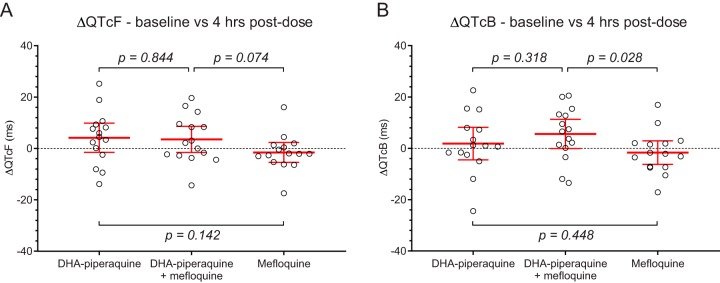
Changes in the electrocardiogram QTcF (A) and QTcB (B) between baseline and 4 h after administration of DHA-piperaquine alone, DHA-piperaquine + mefloquine, and mefloquine alone. Open circles are observed changes in QTc intervals, and solid red lines are mean values ± 95% confidence intervals.

**FIG 2:**
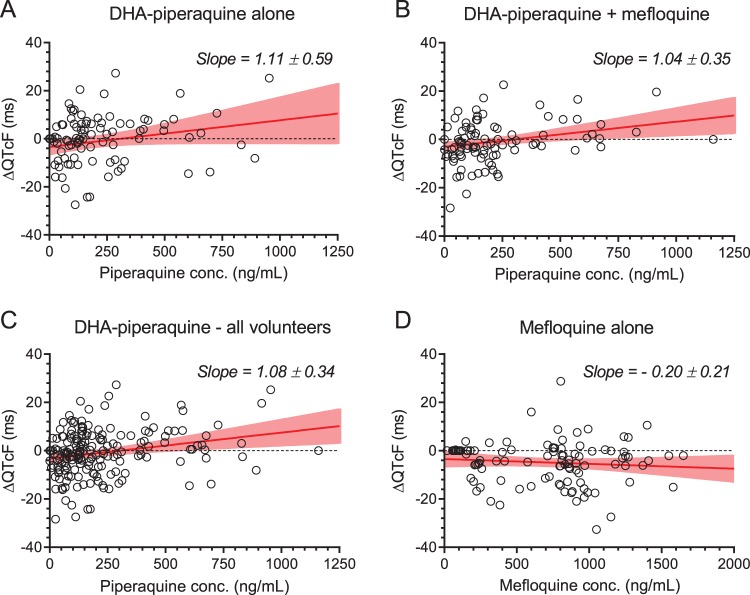
Ordinary linear regression of QTcF interval prolongations (ΔQTcF) versus piperaquine drug concentrations (A, B, and C) and versus mefloquine drug concentrations (D). (A and B) The relationship between piperaquine drug concentrations and ΔQTcF in healthy volunteers receiving DHA-piperaquine alone (A) and DHA-piperaquine + mefloquine (B). (C) Relationship between piperaquine drug concentrations and ΔQTcF for all volunteers receiving DHA-piperaquine (all arms). (D) Relationship between mefloquine drug concentrations and ΔQTcF for volunteers receiving mefloquine alone. Open circles are observed ΔQTcF at specific drug concentrations. Slopes are displayed as mean regression lines (solid red lines) and 95% confidence intervals (shaded area) and as mean values ± standard errors.

In assessment of the QT interval (see Fig. S1 in the supplemental material), Bazett’s correction resulted in a significant overcorrection of QT intervals (*P* < 0.0001), whereas Fridericia’s correction generated no residual trend in the corrected QT intervals versus heart rates. Baseline heart rates, QT interval corrected by Fridericia’s formula (QTcF interval), and Bazett-corrected QT interval (QTcB) were similar before each of the interventions. A maximum increase of the QTcF and QTcB was seen 4 h after administration of both DHA-piperaquine and DHA-piperaquine + mefloquine (Table 3 and Fig. 1). No significant prolongation of either QTc interval was found after administration of mefloquine alone. The mean (standard deviation [SD]) increase of QTcF and QTcB intervals between baseline and hour 4 was not significantly different after DHA-piperaquine and DHA-piperaquine + mefloquine: 4.2 (10.3) ms versus 3.5 (9.2) ms and 1.8 (11.4) ms versus 5.6 (10.3) ms, respectively. QTcF prolongations correlated positively with plasma piperaquine concentrations (Fig. 2), and this relationship was unaffected by the coadministration of mefloquine (Fig. 2A and B). Pooling all piperaquine data resulted in 1.08-ms QTcF prolongation per 100-ng/ml increase in piperaquine concentrations (Fig. 2C). QTcF prolongation did not correlate with mefloquine plasma concentrations (Fig. 2D).

**TABLE 3:** Mean changes compared to baseline in heart rate and QTc interval, stratified by drug regimen^*a*^

Time point	Value(s) for study drug(s)								
	DHA-piperaquine			DHA-piperaquine + mefloquine			Mefloquine		
	ΔHR (bpm)	ΔQTcF (ms)	ΔQTcB (ms)	ΔHR (bpm)	ΔQTcF (ms)	ΔQTcB (ms)	ΔHR (bpm)	ΔQTcF (ms)	ΔQTcB (ms)
H1	2.2 (4.2)	−2.4 (8.2)	−0.3 (9.9)	**3.1 (3.7)**	**−8.2 (7.8)**	**−4.9 (8.3)**	2.1 (3.4)	−4.8 (12.2)	−2.6 (12.2)
H2	0.1 (4.5)	−4 (9.1)	−4 (10.9)	**2.9 (4.0)**	**−4.7 (5.8)**	−1.5 (7.0)	0.8 (3.3)	**−6 (6.0)**	**−5.1 (7.7)**
H4	−2 (4.6)	4.2 (10.3)	1.8 (11.4)	1.7 (3.7)	3.5 (9.2)	5.6 (10.3)	−0.1 (3.1)	−1.6 (7.0)	−1.7 (8.3)
H8	**2.9 (4.8)**	−4 (21.6)	−1.1 (22.3)	**4.2 (3.3)**	−1.4 (8.2)	3.1 (9.5)	**6 (4.8)**	**−10.8 (10.4)**	−4.7 (12.7)
H12	2.6 (6.4)	−5 (16.5)	−2.3 (19.8)	**5.7 (3.1)**	−1.3 (9.7)	4.8 (9.9)	**6.9 (5.3)**	**−7.6 (9.0)**	−0.6 (12.4)

a Changes in heart rate (ΔHR), ΔQTcF, andΔQTcB between baseline and up to 12 h after administration of DHA-piperaquine alone, DHA-piperaquine + mefloquine, and mefloquine alone. Data are presented as mean (SD). Significant changes (by paired *t* test) compared to baseline are indicated in boldface.

### Pharmacokinetic analysis.

**FIG 3:**
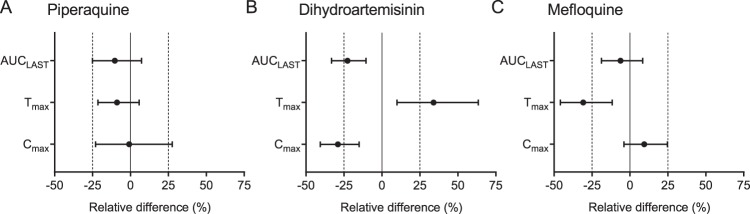
Forest plots showing the geometric mean pharmacokinetic parameter ratios based on 15 individuals (14 for mefloquine) and the corresponding 90% confidence interval for the drugs given alone or in combination with other drugs. AUC_LAST_ represents the area under the concentration-time curve from time zero to the last measurable concentration. *C*_max_ is the maximum concentration, and *T*_max_ is the time to reach the maximum concentration. Solid vertical lines represent no interaction (zero difference), while vertical dashed lines represent a clinically relevant effect of ±25% relative difference. (A) Piperaquine pharmacokinetic parameter ratios when DHA-piperaquine is given alone and in combination with mefloquine. (B) DHA pharmacokinetic parameters when DHA-piperaquine is given alone and in combination with mefloquine. (C) Mefloquine pharmacokinetic parameters when mefloquine is given alone and in combination with DHA-piperaquine.

Pharmacokinetic parameter estimates for dihydroartemisinin, piperaquine, and mefloquine when given alone and in combination are presented in Table 4. Coadministration of mefloquine did not significantly impact the pharmacokinetic properties of piperaquine (maximum concentration of drug in serum [*C*_max_], −0.82% [90% confidence interval, or CI, −22.9, 27.6; *P* = 0.888]; area under the concentration-time curve from administration of the drug until the last detectable drug concentration [AUC_LAST_], −10.3% [90% CI, −25.1, 7.30; *P* = 0.239]; time to maximum concentration of drug in serum [*T*_max_], −8.83% [90% CI, −21.4, 5.77; *P* = 0.301]) (Fig. 3 and Fig. S2). One individual in the mefloquine-alone arm was removed, as he had much lower plasma concentration than the rest of the patients (*C*_max_, after curve stripping, for this individual was 95.0 ng/ml compared to a median of 1,040 ng/ml for the other individuals in this arm). The reason for the very low concentrations are unknown, but this was in the mefloquine-alone arm, so a drug-drug interaction can be excluded. The same participant had normal mefloquine concentrations when given mefloquine with DHA-piperaquine, suggesting normal distribution and elimination properties. Except for a significantly shorter time to peak levels of mefloquine (*T*_max_, −30.8% [90% CI, −45.9, −11.6; *P* = 0.0475]), there was also no significant impact on the pharmacokinetic properties of mefloquine when given with DHA-piperaquine (*C*_max_, 9.44% [90% CI, −3.97, 24.7; *P* = 0.348]; AUC_LAST_, −6.17% [90% CI, −18.8, 8.39; *P* = 0.350]). However, coadministration of DHA-piperaquine and mefloquine did result in a significantly lower exposure to dihydroartemisinin and a longer time to peak levels of dihydroartemisinin (*C*_max_, −29.0% [90% CI, −40.6, −15.1; *P* = 0.0022]; AUC_LAST_, −22.6% [90% CI, −33.1, −10.4; *P* = 0.0039]; *T*_max_, 34.0% [90% CI, 9.87, 63.5; *P* = 0.0079]).

**TABLE 4:** Pharmacokinetic parameter estimates for 15 healthy volunteers stratified by drug regimen^*c*^

Parameter and regimen component	Value(s) by drug regimen			*P* value
	DHA-piperaquine alone	Mefloquine alone^*b*^	Coadministered	
Dihydroartemisinin				
*T*_max_ (h)	1.00 (1.00–2.00)		1.50 (0.500–3.00)	**0.0112**
*C*_max_ (ng/ml)	387 (184–792)		275 (124–510)	**0.0026**
AUC_LAST_ (h·mg/ml)	901 (394–2,000)		673 (360–1,550)	**0.0151**
AUC_inf_ (h·mg/ml)	908 (398–2,030)		684 (365–1,580)	**0.0151**
*t*_1/2_ (h)	2.03 (1.13–2.60)		1.94 (1.06–2.26)	0.0637
CL/F (liters/h)	132 (59.0–302)		176 (75.8–329)	0.0413
V/F (liters)	337 (164–678)		436 (197–846)	0.0946
Piperaquine				
*T*_max_ (h)	4.00 (2.00–4.00)		3.00 (2.00–4.00)	0.438
*C*_max_ (ng/ml)	539 (240–1,040)		631 (229–1,160)	0.668
AUC_LAST_ (h·mg/ml)	17.1^*a*^ (8.11–36.8)		16.8 (7.36–27.7)	0.277
AUC_inf_ (h·mg/ml)	19.8^*a*^ (12.2–58.5)		22.4 (8.81–32.5)	0.966
*t*_1/2_ (h)	12.7^*a*^ (7.35–36.6)		13.7 (6.97–53.0)	0.700
CL/F (liters/h)	25.9^*a*^ (8.79–42.7)		24.7 (17.1–62.9)	0.365
V/F (liters)	9,600^*a*^ (7,500–33,900)		16,100 (4,660–34,300)	0.320
Mefloquine				
*T*_max_ (h)		6.00 (4.00–10.0)	4.00 (2.00–10.0)	0.0679
*C*_max_ (ng/ml)		1,040 (711–1,580)	1,110 (854–1,450)	0.358
AUC_LAST_ (h·mg/ml)		334 (261–596)	295 (232–518)	0.194
AUC_inf_ (h·mg/ml)		431 (342–1,050)	386 (330–1,160)	0.241
*t*_1/2_ (h)		16.4 (12.7–28.6)	19.4 (13.0–32.9)	0.357
CL/F (liters/h)		1.16 (0.475–1.46)	1.29 (0.432–1.51)	0.173
V/F (liters)		638 (438–965)	721 (484–1,160)	0.194

a Based on 15 volunteers. Four volunteers were sampled for 24 h after dose administration and therefore were excluded from this parameter summary but included in the statistical analysis.

b One volunteer was excluded from these parameter summaries and statistical analyses.

c Values are presented as median (min-max). *T*_max_ is the time to reach maximum concentration, *C*_max_ is the maximum concentration, AUC_LAST_ is total exposure up to the last observation, AUC_inf_ is the total exposure extrapolated to infinity, *t*_1/2_ is the terminal elimination half-life, CL/F is the apparent elimination clearance, and V/F is the apparent volume of distribution. The *P* value was obtained from the Wilcoxon matched-pairs signed-rank test.

## DISCUSSION

In extensive clinical use, DHA-piperaquine has proved a well-tolerated and highly effective antimalarial drug. The main clinical concern has been the effect of piperaquine on ventricular repolarization (manifest by electrocardiographic QT prolongation), although recent large studies suggest that this does not translate into an increased risk of lethal ventricular tachyarrhythmias (25). Halofantrine, an antimalarial drug that has now been withdrawn, caused marked QT prolongation and did cause lethal ventricular tachyarrhythmias (28). This effect was accentuated by concomitant exposure to mefloquine. Naturally, there was concern that adding mefloquine to piperaquine could be dangerous, but fortunately there was no evidence of additional QT prolongation. As expected, DHA-piperaquine prolonged the QTcF and QTcB intervals, with a maximum effect at peak levels around 4 h postdose, but the addition of mefloquine did not lead to an increase of QTc (QT interval corrected for heart rate) prolongation. One subject suffered from a moderate-severity neuropsychiatric reaction after DHA-piperaquine + mefloquine, reflecting the known, neuropsychiatric side effects of mefloquine. A recent pooled analysis found the risk of neuropsychiatric events to be 7.6/10,000 treatments (95% CI, 4, 12) (23).

There is a potential for drug-drug interactions between mefloquine and piperaquine, since both drugs are metabolized by cytochrome P450 (CYP) 3A4, which can be inhibited by piperaquine according to *in vitro* studies (29, 30). However, this study showed no clinically relevant interactions. Only the time to reach the maximum concentration showed a significant difference. However, the pharmacokinetic properties of dihydroartemisinin were affected by coadministration of mefloquine, a drug-drug interaction that could be of clinical significance. The total exposure to dihydroartemisinin was 23% lower (90% CI, −33.1, −10.4) when combined with mefloquine. The reason for this is unknown, but it could be a consequence of altered absorption or metabolism/elimination of the drug. Data generated here do not provide any insight into this effect, and further studies are needed to understand the underlying mechanism of this interaction. However, previous studies in healthy volunteers (*n* = 10) and patients (*n* = 207) with uncomplicated falciparum malaria in Thailand demonstrated no pharmacokinetic drug-drug interactions when administering dihydroartemisinin and mefloquine alone or in combination (31, 32). The dose of dihydroartemisinin in the DHA-piperaquine combination is already relatively low (2.25 to 2.50 mg/kg of body weight) compared to that of other ACTs (4 mg/kg), so it will be important to assess if this interaction has clinical relevance in the larger series of patients.

The power of multidrug approaches for the management of infections is evident from high efficacy and the relatively slow emergence and spread of resistance to therapies for HIV and tuberculosis. The chance of a malaria parasite developing resistance to two antimalarials *de novo* is small, provided that the mechanisms are unrelated and not conferred by the same mutation. Combining three antimalarials reduces the risk even further. In the case of triple ACTs, the artemisinins kill a large amount of parasites in the first 3 days of treatment. Any remaining parasites that normally would face only one partner drug would now be exposed to two partner drugs, reducing the chance of treatment failures and providing mutual protection against resistance. In the short term, the combination of DHA-piperaquine-mefloquine could lead to a restored efficacy in areas affected by resistance to piperaquine (eastern GMS) or mefloquine (eastern Myanmar). In the longer term, deployment of TACTs could slow down or prevent the emergence of artemisinin and partner drug resistance.

It is likely that artemisinin resistance will compromise the efficacy of ACTs containing novel partner drugs and will contribute to selection of resistance against partner drugs that are still under development or have only been deployed on a small scale. For example, the artesunate and pyronaridine (Pyramax) combination had failure rates of up to 16.0% and 10.2% in two sites in western Cambodia, an area with a high prevalence of artemisinin resistance, despite high efficacy in other African and Asian countries (33, 34). Therefore, combining any novel antimalarial with two other antimalarials in order to prolong the longevity of novel and existing compounds warrants further consideration.

The wash-out period between each regimen was 6 weeks only and was therefore too short to eliminate the drugs completely. The median (range) mefloquine concentration was 87.9 (17.9 to 179) ng/ml at the end of the 6-week wash-out period. The median (range) maximum concentration when giving mefloquine alone was 1,095 ng/ml (866 to 1,650), without curve stripping, indicating that the predose concentrations contributed approximately 8% to the total peak concentration (median [range] of 7.53% [1.67% to 18.0%]). However, the application of curve stripping corrected for this bias and allowed the pharmacokinetic parameters to be compared when mefloquine was given alone and in combination with DHA-piperaquine. Another limitation was that all subjects were recruited from separate earlier studies, making use of existing DHA-piperaquine-alone arms. Although this design did not allow us to perform a complete study arm randomization, we acknowledge that randomization of the treatment sequence of DHA-piperaquine + mefloquine and mefloquine alone could have helped to minimize the potential confounding carryover effect. However, this design permitted us to reuse available data and, therefore, reduce the duration and cost of the study as well as potential discomfort from drug administrations and sampling in healthy volunteers.

This pharmacokinetic/pharmacodynamic study was conducted according to current European Medicines Agency and U.S. FDA guidance, and we believe that the data generated are important and increase insights into safety of the proposed TACT. However, malaria has been shown to have a substantial impact on the QT interval and may lead to false conclusions on QT prolongation properties of antimalarial drugs. Potential drug-drug interactions might also be somewhat different in malaria patients than healthy volunteers due to the acute disease effect associated with malaria during the first days of treatment. Thus, the findings in this study need to be confirmed in patients with malaria.

## MATERIALS AND METHODS

### Sample size calculation.

The primary focus was potential cardiotoxicity. DHA-piperaquine prolongs the QT interval corrected by Fridericia’s formula (QTcF interval) by a mean of 10 ms (SD, 13 ms) (reanalysis of published data [35]). A sample size of 13 subjects would allow detection of a further increase of 15 ms with a power of 90% and α of 5%. The sample size was adjusted to a total of 16 subjects to allow for loss to follow-up and other unforeseen circumstances. A sample size of 11 (*n* = 11) volunteers would allow a paired *t* test, applied to continuous pharmacokinetic variables (e.g., AUC_LAST_, *C*_max_, and *T*_max_), to demonstrate a significant difference (power of 80% and α of 5%) when the standard deviation of the paired differences is not larger than the actual paired differences.

### Study overview.

Healthy male and female Thai subjects were enrolled in an open-label, sequential, single-dose study of orally administered DHA-piperaquine, DHA-piperaquine + mefloquine, and mefloquine. The study was conducted in the healthy volunteer research ward at the Hospital for Tropical Diseases, Faculty of Tropical Medicine, Mahidol University, Bangkok, Thailand, and was approved by the Ethics Committee of the Faculty of Tropical Medicine (Mahidol University, Bangkok, Thailand) (TMEC 14-069) and the Oxford Tropical Research Ethics Committee (University of Oxford, Oxford, United Kingdom) (58-11). The trial was registered at clinicaltrials.gov (NCT02324738).

### Study subjects.

Clinically healthy males and females, aged between 18 and 60 years, weighing between 36 and 75 kg, and willing to comply with the study protocol for the duration of the trial, were eligible for this study. All gave fully informed written consent. Exclusion criteria were a QTcF interval of ≥450 ms or a history of any cardiac disease or a family history of sudden cardiac death; positive hepatitis B, hepatitis C, or HIV serology; a creatinine clearance of <70 ml/min as determined by the Cockcroft-Gault equation; history of alcohol or illicit substance abuse or dependence within 6 months of the study; use of prescription or nonprescription drugs (excluding paracetamol up to 2 g/day), vitamins, and herbal and dietary supplements within 7 days or 14 days for drugs known to have enzyme-inducing characteristics; participation in a clinical trial and receiving a new chemical entity within 30 days or twice the duration of the biological effect (whichever is longer); unwillingness to abstain from alcohol 48 h before and throughout the study; blood donation in the previous 30 days; a history of allergy to the study drugs; inability to comply with the study protocol; alanine aminotransferase (ALT) or aspartate aminotransferase (AST) levels of >1.5× the upper limit of normal; any history of renal disease, hepatic disease, and/or status after cholecystectomy; and antimalarial use in the previous 3 months. Also excluded were female subjects of child-bearing potential who could not comply with the use of effective methods of contraception during the study period until the end of the follow-up period, those who had a positive urine pregnancy test, and those who were lactating.

### Study drug administration and study procedures.

All subjects had participated in previous healthy volunteer studies in which they took 3 tablets of DHA-piperaquine (40/320 mg/tablet; Sigma-Tau) without any other medication (ClinicalTrials registration no. NCT01525511 and NCT02192944) (24, 35). The data obtained from that treatment round were used as a comparator for this study. The subjects were treated in 2 sequential rounds with the combination of DHA-piperaquine (3 tablets; 40/320 mg/tablet; Sigma Tau) and mefloquine (2 tablets; 250 mg base/tablet; Mequine; Thai Government Pharmaceutical Organization, Bangkok, Thailand), followed by mefloquine alone (2 tablets; 250 mg/tablet; Mequine). The two dosing rounds were separated by a wash-out period of at least 6 weeks. For every treatment round, the study drugs were administered under direct observation as a single oral dose after a standard light meal (a small cup of Thai-style porridge with chicken breast, around 200 calories, with less than 50% of the calories from fat) followed by 4 h of fasting. Fluids were restricted to 3 liters/day during the 24 h after the drug dosing. The use of illicit drugs and the intake of grapefruit or grapefruit juice was not allowed throughout the study periods. Alcohol and caffeine drinks were not allowed within 48 h prior to the study drug administration and during the admission. All subjects were admitted to the healthy volunteer research ward for a total of 2 nights and 1 day during each treatment round of the study for the clinical and pharmacokinetic evaluations. Medical history was documented, and a physical examination was performed by the study physicians before, during, and after the study. Blood tests, consisting of a complete blood count, and measurements of fasting blood sugar (FBS), serum creatinine, blood urea nitrogen, serum alkaline phosphatase, ALT, AST, total and direct bilirubin, creatine kinase, potassium, and sodium were performed at screening, at baseline, and 24 h after each drug dose. For women, a serum or urine pregnancy test was performed before each drug administration, and use of contraception was advised throughout the study period and for 4 weeks after the last dose of drugs to prevent pregnancy while using this new drug combination. An electrocardiogram was performed at baseline and at 1, 2, 4, 8, 12, and 24 h after drug dosing (ECG-1250 Cardiofax S; Nihon Kohden, Tokyo, Japan). The QTc (QT interval corrected for heart rate) is calculate by using Fridericia’s correction [QTcF = QT interval/3√(60/heart rate)] and Bazett’s correction [QTcB = QT interval/√(60/heart rate)]. Symptom questionnaires, assessing the presence and severity (mild, moderate, severe, or life-threatening) of symptoms, were taken at baseline and 1, 4, and 24 h and 3 and 7 days after administration of the treatments with DHA-piperaquine + mefloquine and mefloquine. This standard questionnaire was not used in the earlier studies. Adverse events were captured and graded according to the Division of AIDS table for grading the severity of adult and pediatric adverse events, version 1.0, December 2004, with clarification in August 2009 (36).

### Pharmacokinetic study sample collection.

Blood samples for drug plasma concentration measurements were taken at 0.25, 0.5, 1, 1.5, 2, 3, 4, 6, 8, 10, 12, and 24 h and 3, 4, 7, 11, 15, 22, and 36 days after administration of the study drugs. All blood samples were obtained through an indwelling venous catheter during the first 24 h and by venipunctures at later time points. Blood samples were collected in fluoride-oxalate tubes (2 ml). Whole-blood samples were centrifuged for 7 min at 2,000 × *g* at 4°C to obtain plasma for DHA, mefloquine, and piperaquine concentration measurements. Plasma samples were stored immediately at −70°C or lower in a non-self-defrosting freezer until analyzed. All samples were transferred to the Department of Clinical Pharmacology, Mahidol-Oxford Tropical Medicine Research Unit, Bangkok, Thailand, for drug measurements.

### Safety analysis.

All subjects who received at least 1 dose of the study drug were included in the safety analysis. The safety and tolerability of DHA-piperaquine, mefloquine, and the combination of these 3 drugs were assessed by reporting the incidence of adverse events (AEs) and serious adverse events (SAEs) and comparison of the prolongation of QTcF and QTcB intervals for all study arms. QTcF and QTcB intervals were calculated using the QT interval and heart rate, as measured by the electrocardiogram machine. Statistical analysis of safety-related endpoints was performed using Stata v15.0 (StataCorp, College Station, TX, USA).

### Drug analysis.

Concentrations of piperaquine, dihydroartemisinin, and mefloquine were measured using methods validated according to U.S. FDA guidelines (37, 38). Drug quantification was performed in a quality-controlled setting using liquid chromatography coupled with tandem mass spectrometry. In brief, plasma sample preparation was performed in a 96-well format on a Freedom EVO liquid handler system (Tecan, Männedorf, Switzerland) using an MPC-SD SPE plate (3M, Eagan, MN, USA) for piperaquine, Oasis HLB μElution SPE plate (Waters, Milford, MA, USA) for DHA, and a Phree phospholipids removal plate (Phenomenex, Torrance, CA, USA) for mefloquine. Stable isotope-labeled internal standards were used to compensate for recovery and matrix effects. The extracted drugs were separated using a Dionex Ultimate 3000 UHPLC (Thermo Fisher Scientific, Waltham, MA, USA) equipped with a Gemini C_18_ column (Phenomenex) for piperaquine, a Hypersil Gold C_18_ column (Thermo Fisher Scientific) for DHA, and a Zorbax SB-CN (Agilent, Santa Clara, CA, USA) for mefloquine. An API500 triple-quadrupole mass spectrometer and Analyst 1.7 software (ABSciex, Framingham, MA, USA) were used for drug detection and quantification. The coefficient of variation for the quality control (QC) samples was within the set limits and did not exceed 10% for any QC level. The lower limits of quantification were 1.96 ng/ml for DHA, 9.55 ng/ml for mefloquine, and 1.5 ng/ml for piperaquine.

### Pharmacokinetic and pharmacodynamic analysis.

A noncompartmental analysis, as implemented in the software Phoenix 64 (Certara, Princeton, NJ, USA), was performed to evaluate potential pharmacokinetic interactions of dihydroartemisinin, piperaquine, and mefloquine. Noncompartmental analyses were performed for all arms in the study. The observed concentrations were used to derive the maximum concentration (*C*_max_) and the time to reach the maximum concentration (*T*_max_). The drug exposure, measured as area under the concentration-time curve from administration of the drug until the last detectable drug concentration (AUC_LAST_), was derived using the trapezoid method. Linear interpolation was used for ascending concentrations and log-linear interpolation for descending concentrations. The terminal elimination half-life (*t*_1/2_) was estimated by ln2/λ, where λ is the terminal elimination rate constant, estimated from the log-linear best-fit regression of observed concentrations in the elimination phase. The terminal elimination rate constant was used to extrapolate AUC_LAST_ from the last observed concentration to infinity (AUC_inf_ = *C*_LAST_/λ). The apparent elimination clearance (CL/F) and apparent volume of distribution (V/F) were calculated according to standard equations. Curve stripping was applied in the mefloquine-alone arm, as the wash-out period was not long enough to eliminate completely the mefloquine administered in the previous arm (mefloquine given together with DHA-piperaquine). Four individuals had samples collected for 24 h only after administration of DHA-piperaquine. Exposure to piperaquine for up to 24 h after dose (AUC_24_) was estimated for these volunteers, and corresponding piperaquine data were truncated at 24 h in these volunteers when receiving DHA-piperaquine-mefloquine. The individual AUC_24_ values were compared between treatment arms and included in the overall statistical evaluation of drug-drug interactions for piperaquine. Wilcoxon matched-pairs signed-rank tests were performed in GraphPad Prism to compare pharmacokinetic parameters when given alone and in combination with concomitant treatment. The geometric means (with 90% confidence interval) of the ratios of *T*_max_, *C*_max_, and AUC_LAST_ when given alone and in combination with other drugs were evaluated using the bioequivalence function in Phoenix 64 and were plotted in GraphPad Prism, v 8.1 (GraphPad Software Inc., CA, USA).

Ordinary linear regression analysis (GraphPad Prism, v 8.1) was used to quantify the relationship between drug concentrations and prolongation of QTcF interval, and the mean value and 95% confidence interval of the slope of the regression were compared between arms.

## Supplementary Material

Supplemental file 1
