# Supplementary material for: Sequential Open-Label Study of the Safety, Tolerability, and Pharmacokinetic Interactions between Dihydroartemisinin-Piperaquine and Mefloquine in Healthy Thai Adults
Source: Antimicrob Agents Chemother. 2019 Jul 25;63(8):e00060-19. doi: 10.1128/AAC.00060-19 (PMC6658739; doi:10.1128/AAC.00060-19)
Supplement: Supplemental file 1 [file AAC.00060-19-s0001.pdf]

Supplementary material

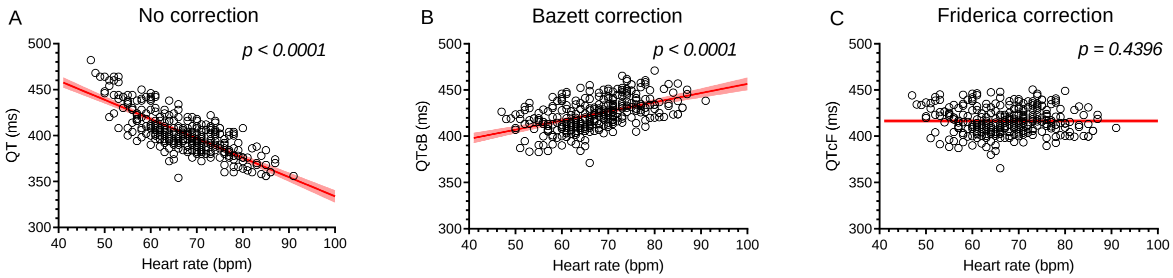

Figure S1. Ordinary linear regression of observed QT intervals and heart rates, when applying (A) no correction, (B) Bazett's correction, and (C) Fridericia's correction. Open circles are observed QT(c) intervals at specific heart rates. Slopes are displayed as mean regression lines (solid red lines) and 95% confidence intervals (shaded areas), and p-values demonstrate if the regression line deviates significantly from zero.

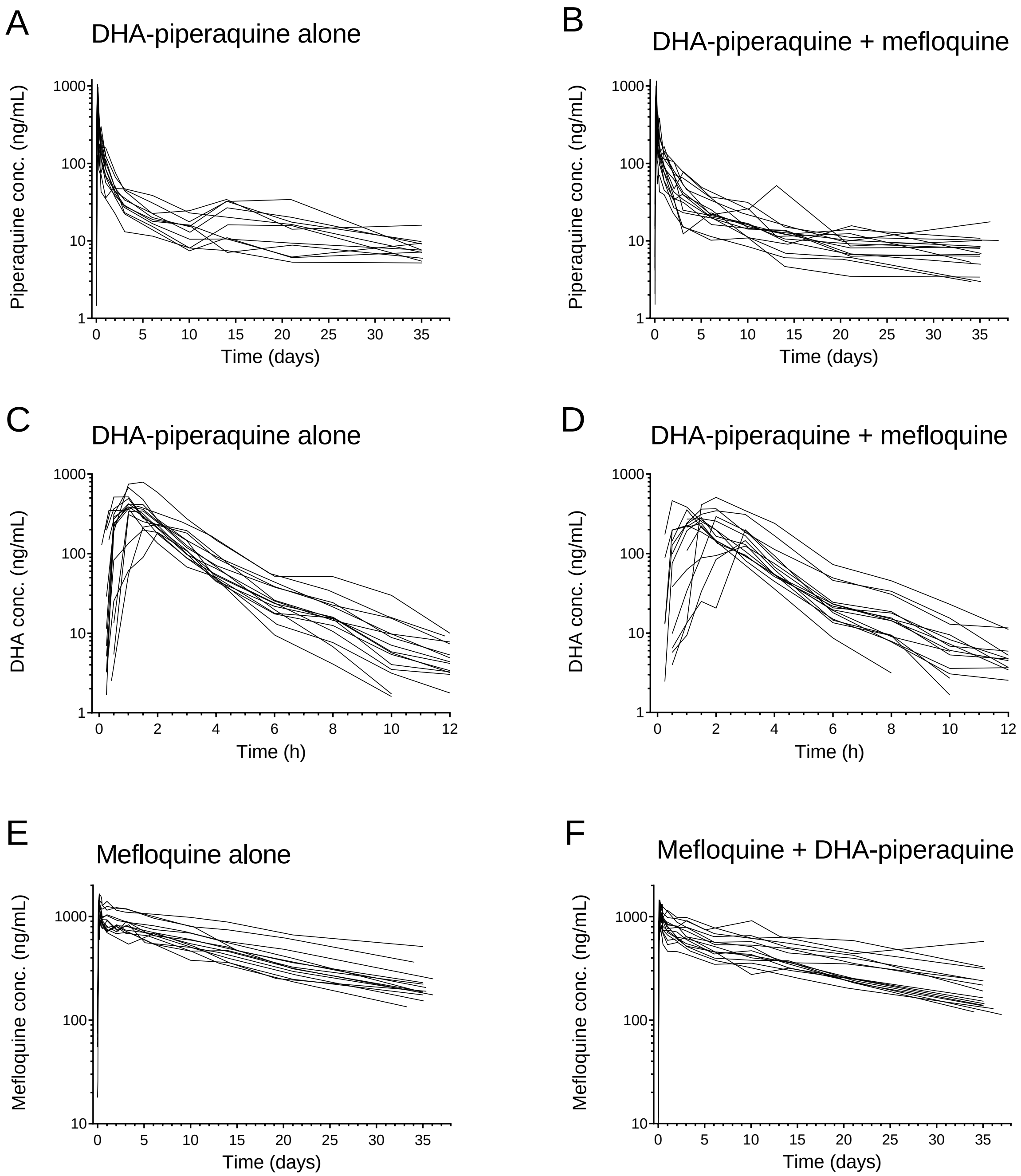

Figure S2. Concentration-time profiles of dihydroartemisinin (n=15), piperaquine (n=15), and mefloquine (n=14), when administered alone and in combination. Top panels show piperaquine concentration-time profiles when administered as DHA-piperaquine alone (A) and together with mefloquine (B). Middle panels show dihydroartemisinin concentration-time profiles when administered as DHA-piperaquine alone (C) and together with mefloquine (D). Bottom panels show mefloquine concentration-time profiles when administered as mefloquine alone (E) and together with DHA-piperaquine (F).
